# Well-Being and Social Capital on Planet Earth: Cross-National Evidence from 142 Countries

**DOI:** 10.1371/journal.pone.0042793

**Published:** 2012-08-15

**Authors:** Rocío Calvo, Yuhui Zheng, Santosh Kumar, Analia Olgiati, Lisa Berkman

**Affiliations:** 1 Department of Global Practice, Graduate School of Social Work, Boston College, Chestnut Hill, Massachusetts, United States of America; 2 Harvard Center for Population and Development Studies, Cambridge, Massachusetts, United States of America; 3 Institute for Health Metrics and Evaluation, Department of Global Health, School of Public Health, University of Washington, Seattle, Washington, United States of America; 4 Department of Society, Human Development, and Health, Harvard School of Public Health, Boston, Massachusetts, United States of America; 5 Department of Epidemiology, Harvard School of Public Health, Boston, Massachusetts, United States of America; 6 Department of Global Health and Population, Harvard School of Public Health, Boston, Massachusetts, United States of America; Tulane University, United States of America

## Abstract

High levels of social trust and social support are associated with life satisfaction around the world. However, it is not known whether this association extends to other indicators of social capital and of subjective well-being globally. We examine associations between three measures of social capital and three indicators of subjective well-being in 142 low-, middle- and high-income countries. Furthermore, we explore whether positive and negative feelings mirror each other or if they are separate constructs that behave differently in relation to social capital. Data comes from the Gallup World Poll, an international cross-sectional comparable survey conducted yearly from 2005 to 2009 for those 15 years of age and over. The poll represents 95% of the world's population. Social capital was measured with self-reports of access to support from relatives and friends, of volunteering to an organization in the past month, and of trusting others. Subjective well-being was measured with self-reports of life satisfaction, positive affect, and negative affect. We first estimate random coefficient (multi-level) models and then use multivariate (individual-level) Ordinary Least Square (OLS) regression to model subjective well-being as a function of social support, volunteering and social trust, controlling for age, gender, education, marital status, household income and religiosity. We found that having somebody to count on in case of need and reporting high levels of social trust are associated with better life evaluations and more positive feelings and an absence of negative feelings in most countries around the world. Associations, however, are stronger for high- and middle-income countries. Volunteering is also associated with better life evaluations and a higher frequency of positive emotions. There is not an association, however, between volunteering and experiencing negative feelings, except for low-income countries. Finally, we present evidence that the two affective components of subjective well-being behave differently in relation to different indicators of social capital and social support across countries.

## Introduction

High subjective well-being (SWB) is associated with many desirable outcomes such as positive development among young adults [1], healthier and longer lives [2] and democratic attitudes [3]. Research suggests that social capital and subjective well-being are correlated. Evidence shows that social trust and social support are associated with life satisfaction globally and that the correlation is stronger in high-income countries [4], [5]. Concerning the affective component of subjective well-being, having somebody to count on in case of emergency is associated with experiencing positive emotions across nations [6], [7].

Most of this research has been conducted in relation to the association between the cognitive component of subjective well-being, life satisfaction, and social trust and social support. Both subjective well-being and social capital are multidimensional constructs. In this paper, we are interested in exploring whether the cognitive (eudaimonic) and affective (hedonic) components of subjective well-being are similarly associated with social trust, social support and volunteering around the world. In addition, we hope our evidence will help to clarify the highly inconsistent literature on the relation between the two hedonic components of subjective well-being [8] by testing whether positive and negative feelings are polar opposites or whether they behave differently in relation to social support and social capital proxies. No research has examined this question systematically employing a sample of the world's population.

### Evidence on the Relationship between Social Capital and Subjective Well-Being

Despite substantial post-war economic growth, North Americans and British are neither happier nor more satisfied with their lives nowadays than they were a quarter of century ago [9]. A similar trend has been observed in China where people are less satisfied with their lives than they were before the astounding economic progress experienced over the last 30 years [10]. The fact that above a modest threshold, greater wealth does not contribute to individuals' well-being is well established in the literature [11], [12] (see Stevenson and Wolfers [13] for a challenge to this assumption). The question then is *what does contribute to well being*? Recent evidence suggests that social capital may be a good candidate.

Studies conducted with data from the Gallup World Poll suggest that living in a trustworthy environment and having relatives and friends to count on in case of need are consistently associated with higher levels of life satisfaction worldwide. The relationship between social capital and well-being, however, it is not uniform across countries, it is tighter in high-income nations than in other parts of the world [4], [5], [14], [15].

Analyses of different waves of the World Values Survey have yielded similar results: those who reported the highest levels of subjective well being –measured as self-reported life satisfaction and happiness- had in common frequent visits to family, friends and neighbors, participation in community organizations and residence in high-trust environments [16]. At the national level, high average levels of generalized trust, civic participation and lack of corruption are stronger predictors of life satisfaction than income or economic uncertainty; although the association is clearer for Western societies, particularly for Northern Europe, than for less developed societies [17]. Not every study, however, finds a strong global association between social capital and subjective well-being. A recent cross-national study of 95 countries from the World Values Survey shows a positive, although fragile, relationship between self-reported satisfaction with life and generalized trust. Furthermore, people in countries representative of transition economies seem to be particular dissatisfied with their lives while the opposite is true for people living in Latin American countries [18].

Alternative evidence with smaller samples and different measures also suggests a positive association between social capital and subjective well-being. In Asia, a study conducted involving 5 countries examining the relationship between social capital and life satisfaction discovered that, adjusting for SES, lacking somebody to discuss important matters with, mistrust in social and political institutions, and reporting lower levels of interpersonal trust were all associated with less life satisfaction. Membership in voluntary organizations, however, was not significantly related to people's well-being [19]. Results from Yip and colleagues' [20] study in rural China also revealed that while both individual and village-level trust correlated with self-reported life satisfaction, belonging to social organizations –including those sponsored by the Communist party- did not. However, a study conducted in Seoul, South Korea, showed that individuals who participated in one or more organizations were more satisfied with their lives than individuals who did not participate. Furthermore, social participation was also associated with self-reported life satisfaction at the ecological level. The other social capital indicator positively associated with subjective well-being, both at the individual and ecological level, was having somebody to lean on in times of trouble [21].

In Colombia, membership in civic associations and perception of reciprocity and trust among group members was not only related to individuals' positive assessment of their lives but it buffered the perception of insecurity in conflict areas [22]. Adjusting for socio-economic indicators both at the individual and at the ecological level, people who trusted their neighbors and who perceived that help was available in case of need reported the highest levels of life satisfaction in the economically deprived suburbs of the Eastern Cape province of South Africa [23], [24].

In Europe, a study examining individual and contextual determinants of life satisfaction in Belgium found that, even adjusting for optimism, higher levels of generalized trust and strong social ties were both related with higher life satisfaction at the individual level. At the ecological level, people who lived in communities with both high rates of unemployment and violent crimes expressed lower well-being [25]. In Germany, people who attended cultural events and church services, who engaged in active sports, who visited friends, relatives or neighbors and who engaged in voluntary work in political and social organizations were more satisfied with their lives than people who did not participate in those activities [26].

Another study conducted with the cycle 17 of the Canadian General Social Survey (GSS17) revealed a clear link between life satisfaction and two proxy measures of social capital, social trust and frequency of visits with family and friends. More specifically, those who reported high levels of trust across a variety of life domains- co-workers, neighbors, and the police- were almost 20% more satisfied with their lives than less trusting individuals. In contrast, associational membership was not significantly related to subjective well-being [5].

### Subjective Well-Being and Social Capital: Concepts and Measurements

Evidence suggests that social trust and social ties are correlated with life satisfaction around the world albeit the relationship is stronger in high-income countries. The relationship between subjective well-being and civic participation however, does not follow a consistent global pattern.

It is important to keep in mind, however, that life satisfaction is only a partial representation of subjective well being -a complex concept that lacks universal definition- but which is often understood as a personal assessment of one's life comprised of two components: (1) a long-term cognitive dimension -life satisfaction- and (2) a temporal affective dimension -positive affect, and low levels of negative affect [27], [28], [29].

Although both components are related with subjective well-being, the correlation is stronger for the cognitive dimension [30]. This empirical evidence aligns with the fact that research has favored the cognitive dimension of subjective well-being because it has traditionally been considered a more stable indicator than the affective dimension. The cognitive dimension of subjective well-being is related to the eudaimonic philosophical tradition which entails the realization of one's potential in accordance with one's true nature. It therefore involves an evaluative component of one's own well-being and it is considered less susceptible to external circumstances. In contrast, the affective dimension of subjective well-being relates to the hedonic philosophical tradition and stresses immediateness and descriptions of emotional states which are more prone to fluctuate [31].

Even though social capital has become a household concept across social science disciplines, a uniform definition has been elusive. From the North American tradition, social capital is understood as a collective property based on relationships. It is traditionally captured using proxy measures of generalized trust, norms of reciprocity and networks in the voluntary sphere among others [32], [33]. The European tradition also considers social networks and connections as a fundamental component of social capital; however, it pays particular attention to the exchange of social support within the networks: ‘contacts and group memberships which, through the accumulation of exchanges, obligations and shared identities, provide actual or potential support and access to valued resources’ [34].

A legitimate concern when studying self-reported constructs such as well-being and social capital is whether they measure what they are supposed to asses (validity) and whether they yield consistent results (reliability).

Concerning validity, a fair body of literature shows that, net of socioeconomic and demographic factors, higher subjective well-being consistently correlates with better objective health outcomes [35] such as low blood pressure [36] or better heart rates [37]. The cross-cultural differences in the concept of well-being [38] are not substantial enough to threaten the validity of the subjective well-being measures across nations [39], [40]. In fact, global evidence reveals that most of variability on subjective well-being is explained by the same objective measures across nations. Alternative life evaluation measures correlate very similarly with social capital proxies around the world [15], [41], [42].

The reliability of the well-being measures is high. Correlations range between 0.6 and 0.7- and it has been gauged by a variety of methods such as posing the same question twice during the same interview, comparing alternative measures of subjective well-being, and with time-series and longitudinal studies looking at the test-retest correlations between responses [5]. Diener and colleagues [39] provide an exceptional review of subjective well-being measures and warn against the use of single-item scales when seeking a finely differentiated understanding of an individual's subjective well-being. Evidence from the Gallup World Poll and from the European Social Survey show that the ecological level the reliability of subjective well-being measures is also high –between 0.88 and 0.98. One reason is that individual-level variations are averaged away and another is that changes in life circumstances are modest over short periods of time [43].

The validity and reliability of the proxy measures for social capital is still in its infancy. Reeskens and Hooghe [44] found a three-item scale of social trust reliable and valid for cross-cultural research on Europe. Another study for 51 countries from the latest World Values Survey supports the validity of the social trust measures across nations after observing that most people understands “trust in out-groups” when asked whether most people can be trusted [45]. More research is needed investigating the validity and reliability of other proxies of social capital across countries.

### Subjective Well-Being and Social Capital: Questions, Theory and Hypotheses

Not until very recently have researchers begun to investigate both aspects of subjective well-being. Worldwide evidence shows that indeed they have different correlates. Income and wealth have a stronger association with the cognitive component than with the affective component of subjective well-being. In contrast, having somebody to count on in case of emergency is closely associated with experiencing pleasant emotions [6], [7], [46]. In the US, Kahneman and Deaton [47] observe that income is both related to life evaluation and emotional well-being. However, whereas life-evaluation rises steadily with income, there is a satiation point for emotional well-being.

Income and social support seem to relate differently with the evaluative and with the emotional components of well-being. We do not know, however, if social trust and volunteering are different correlates of the emotional component of subjective well-being. It also remains to be seen if positive and negative feelings mirror each other or if they are separate concepts that behave differently in relation to social capital. We explore these questions in this paper.

Based on the above evidence we would anticipate a global positive relationship between experiencing positive feelings and having somebody to count on in case of emergency. Life satisfaction, social support and generalized trust will be also positively associated around the world, –although the correlation will be stronger in high-income countries. One interpretation is that social capital is unequally distributed among nations and that wealthier countries have higher social capital endowments; higher social trust and more social relations [48]. Another interpretation is that the economic prosperity experienced by wealthy countries in the last few decades, coupled with the creation of the welfare state, has propitiated a change of paradigm from materialistic to postmaterialistic values where concerns of belonging and participation in society predominate over the uneasiness of economic insecurity [49]. A study conducted with the last wave of the World Values Survey for 48 countries shows that this is indeed the case after finding a consistent pattern towards post-materialist life satisfaction when moving from poor to richer countries [50]. This argument is in line with Maslow's theory of the hierarchy of needs, which anticipates that the relative importance of income and social factors such as friendship and trust would differ between richer and poorer countries [51]. Recent evidence shows a consistent pattern towards postmaterialism; citizens who live in countries where their basic needs are fulfilled report higher life evaluations [7]. A third explanation is that freedom and democracy, two factors closely related to life-satisfaction, are normative in wealthier countries. Most dissatisfied people in the world in the 1990s did not live in the poorest countries but in ex-Communist societies [52].

It is harder, however, to anticipate a global pattern between the third proxy measure of social capital, volunteering, and subjective well-being. It is fairly well-established that engaging in prosocial behavior is related to well-being in many developed countries [53], [54]. Evidence even suggests a causality mechanism after a number of studies showed that people who performed random acts of kindness for a period of time were happier than those in the control group [55], [56]. However, it is not known whether this association extends to less economically advanced countries.

## Methods

### Data

Data come from the Gallup World Poll [57], which began in 2005. Data were collected annually from randomly selected, nationally representative samples in 150 countries, - representing 95% of the world's adult population. Starting in 2005, the survey has annually sampled around 1000 individuals from each country using a standard set of core questions that have been translated into the major languages of the respective country. Not all countries were sampled every year and until 2008, only 78 countries were sampled in all three waves. In contrast to previous international surveys, the Gallup World Poll covers more poor countries in Sub-Saharan Africa and is nationally representative for a larger number of countries. For this study, we used information from data collected during 2005–2009.

### Gallup World Poll Methodology

The target population for the Poll was the entire civilian, non-institutionalized population, aged 15 and older. Telephone surveys, employing Random-Digit-Dial (RDD), were used in countries where telephone coverage represents at least 80% of the population or is the customary survey methodology. In countries were face-to face surveys were conducted; a multistage stratified sampling procedure was adopted. Primary Sampling Units (PSUs), consisting on clusters of households were sampled at first stage, and then stratified by population size and/or geography. Further details of the sampling frame and survey protocols are provided in the Gallup Annual Report [58].

### Sample: The Gallup World Poll

The Poll contained information on 455,104 individuals in 154 countries. In this study we restricted the sample to countries for which information on household income and education was available from 2005 to 2009 (310,891 individuals nested in 149 countries). Further exclusions of observations with missing values for the variables of interest resulted in a sample of 142 countries and 214, 966 individuals. For the analyses involving social trust at the outcome measure, only available for the year 2009 and for 66 countries, the sample size was 56, 561 individuals.

### Measures

The Gallup Poll contains a rich set of measures on subjective well-being. We analyze, in this study, global life evaluation and positive and negative feelings (cognitive and affective indicators of subjective well-being respectively).

The global life evaluation indicator (GLE) is measured using a Cantril's Self-Anchoring Scale, which asked respondents to evaluate their present life in a ladder scale from 0 to 10, with 0 representing the worst possible life and 10 the best possible life. The positive feelings score (PFS) was based on the following two questions: *1) Did you smile or laugh a lot yesterday? 2) Did you experience enjoyment during a lot of the day yesterday?* Response items for both items were “yes”, “no”, “do not know”, and “refused”. The positive feeling score represented the number of “yes” answers to the above questions. It ranged from 0 to 2. The negative feelings score (NFS) corresponds to the number of “yes” responses to four questions on negative feeling experiences a lot yesterday: worry, sadness, depression, and anger. It ranged from 0 to 4. For both affective scores the “do not know” and “refused” responses were negligible. We treated them as missing in the analyses which did not change the results.

The correlations between the three measures were moderate: −0.37 between (PFS) and (NFS), 0.22 between (PFS) and (GLE), and −0.18 between (NFS) and (GLE).

Social support, volunteering and social trust were used as independent variables and were included separately in the analyses. Social support was measured by asking respondents *“if you were in trouble, do you have friends and relatives you can count on to help you whenever you need them, or not.”* Volunteering was measured by asking respondents *“Have you volunteered your time to an organization in the past month.”* We recoded responses to both questions as a binary variable, with “yes” being 1 and 0 otherwise. Social trust was measured by asking *“Do you think people can be trusted or not”*. Approximately 1% of the respondents had either “refused to answer” or “do not know”, and their exclusion did not alter the results.

Social support, volunteering, and social trust were weakly correlated. Among the subsample of individuals in 66 countries with all three measures, the correlation coefficients were 0.04 between social support and volunteering, 0.05 between social support and trust, and 0.07 between volunteering and trust.

We include age, gender, religiosity, marital status, education, and household income (logarithm scale) as covariates that may have an impact on subjective well-being or confound the associations between key independent variables and subjective well-being. Age and income were entered as continuous variables whereas gender, religiosity and marital status were entered as binary variables. The Gallup Poll reports education in three categories: 0–8 years of schooling; 9–15 years of schooling; and four years of education beyond high school. Zero household income was replaced with small positive value to have a meaningful log value. Religiosity was defined as the extent to which respondent considered religion as an important part of their life.

### Analysis

We first estimated a random coefficient model (multi-level) with individuals at the first level and countries at the second level to explore whether the relationship between subjective well-being, social support and social capital varied systematically across countries. At a second stage we implemented country-specific analyses using multivariate ordinary least squares (OLS) with country fixed effects and robust standard errors to correct for heteroskedasticity of the error terms. We estimated OLS even though we acknowledge that the PFS and NFS outcome variables are discrete in nature with limited number of categories. We employ linear models because evidence supports the consistency of the results for linear model estimators with discrete outcomes with large sample sizes and weak assumptions [59]. Moreover, the results from the random coefficient models and the standardized coefficients of the multivariate OLS regressions are easily interpretable and can be compared across outcomes. Finally, previous research with the Gallup Poll data has employed a similar strategy to facilitate comparability across outcomes and countries [6], [47].

We standardize each SWB measure to a have mean of zero and standard deviation of one so that the coefficients can be interpreted as number of standard deviations and can be compared across outcomes.

The random coefficient model is shown in equations (1) and (2).

Level 1: 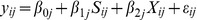
(1)



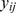
: dependent variable (PFS, NFS, or GLE) for individual 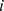 in country 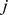



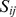
: individual level social capital measure


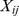
: other individual level covariates


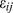
: individual level random component

Level 2:
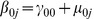
(2)

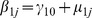


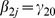




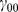
, 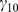 and 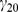 are the fixed components, while 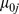 and 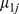 are the country-level random components.

For each standardized SWB measure, we first estimated an intercept-only multilevel linear model, and calculated the intraclass correlation coefficient (ICC) to examine what proportion of total variance was attributed to country-level random variations. The full-scale multilevel model includes one social capital indicator as well as other socio-demographics as covariates. Both the intercept and the coefficient for the social capital measure had a fixed component and a component varying across countries randomly. If the variance for the random component of the coefficient was statistically greater than zero, there was evidence that the association between SWB and the social capital measure varied across countries. Other covariates in the model included age, gender, education, marital status, household income, and interview year. Their coefficients only had the fixed components. Next we conducted OLS regressions with country fixed effects, with the same set of covariates and sample stratification. The regression equation is shown in equation (3).
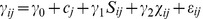
(3)



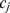
: country-fixed effect


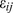
: individual level error term

In treating country-specific effects as fixed effects, we allowed for potential correlations between country-specific effects and covariates. Adding country-specific fixed effects also meant that we focused on within-country associations between SWB and social capital indicators.

Analyses were carried out for all countries combined and then by national income level, according to the World Bank income classification of countries. Subsequently, we examined the association between social support, volunteering or social trust and SWB in each country separately. All analyses were conducted in STATA SE, version 11 (StataCorp, college station, TX, USA). For OLS with country fixed effects or country-specific analyses, data was weighted by individual-level sampling weights provided by Gallup to ensure nationally representative samples in each country.

## Results

### Descriptive Statistics

Descriptive statistics for the outcome variables (SWB measures) explanatory variables and covariates are summarized in Table 1. For the pooled sample, the average PFS is 1.37, meaning the average number of positive feelings is 1.37, out of a maximum of 2; the average NFS is 0.90 (maximum is 4), while the average GLE is 5.20 (maximum is 10). For high-income countries the average PFS is 1.46, and the average GLE is 6.46. For low-income countries these scores are 1.32 and 4.38 respectively. The average NFS ranges from 0.97 in lower-middle income countries to 0.8 in low- income countries.

**Table 1:** Summary statistics of subjective well-being measures, social capital indicators and socio-demographic variables.

Variables	All countries	By national income category			
		High income	Upper middle	Lower middle	Low
**Subjective well-being**					
Positive feelings score (PFS)	1.37	1.46	1.37	1.36	1.32
Negative feelings score (NFS)	0.90	0.87	0.94	0.97	0.80
Life evaluation (GLE)	5.20	6.46	5.44	4.98	4.38
**Social capital (%)**					
Social support	78%	89%	83%	74%	69%
Volunteering	21%	23%	17%	20%	23%
Social trust	22%	25%	20%	19%	25%
**Socio-demographic**					
Mean age (years)	38	44	41	37	34
Female (%)	51%	50%	52%	50%	51%
Married (%)	53%	59%	46%	53%	55%
High school (%)	47%	60%	56%	45%	35%
College (%)	9%	18%	11%	8%	4%
Religiosity (%)	76%	49%	68%	85%	90%
Mean household income (dollars)	14,341	40,247	11,585	7,998	4,772

Data source: Gallup World Poll, 2005–2009. Data is weighted by cross-sectional sampling weights.

Seventy eight percent of the respondents said that they had someone to count on in time of need, our social support measure. By income level, this percentage was highest among high-income countries (89%) and lowest among low-income countries (69%). Twenty-one percent reported doing volunteer work last month. In both low-income and high-income countries, 23% reported doing voluntary work, while in upper middle income countries only 17% reported doing so. Twenty-two percent said that people could be trusted, and the percentages were higher in high- and low- income countries relative to middle-income countries.

In the pooled sample, over half of the respondents are female (51%) and married (53%). The mean age of the sample is 38 years. The mean income is about $14,341. About 76% of the sample reported that religion is an important part of their daily life. About 47% completed more than elementary education but less than a four-year college degree and only 9% are college graduates.

### Multilevel analyses

Table 2 shows the estimated ICCs and variances for the country-level random components of multilevel models, for all countries combined and by national income group. ICCs for PFS and NFS are both quite low, below 0.10, indicating that country-level variations do not contribute much to the total variances of PFS and NFS. ICCs for GLE are larger - 0.24 for the pooled sample and ranging from 0.09 to 0.16 by income group. Models 1 to 3 contain one of the social capital measures (social support, volunteering, and social support) and other control variables. We found that, except for one coefficient (social trust on GLE in upper middle income countries), random coefficients for social capital measures have variance statistically greater than zero, indicating that there are variations across countries regarding the associations between SWB and social capital measures.

**Table 2:** Intra-class correlation coefficients (ICCs) and variances of country level random components in multilevel linear regressions.

		Social Support		Volunteering		Social Trust	
	Intercept-only model	Random intercept	Random coefficient	Random intercept	Random coefficient	Random intercept	Random coefficient
**Positive feelings score**							
All countries	0.07	0.074*	0.018*	0.063*	0.007*	0.082*	0.010*
High income	0.04	0.042*	0.024*	0.036*	0.008*	0.052*	0.010*
Upper middle	0.08	0.104*	0.012*	0.077*	0.005*	0.128*	0.011*
Lower middle	0.09	0.108*	0.012*	0.090*	0.008*	0.111*	0.014*
Low	0.05	0.047*	0.010*	0.044*	0.006*	0.049*	0.006*
**Negative feelings score**							
All countries	0.04	0.068*	0.021*	0.044*	0.007*	0.059*	0.019*
High income	0.03	0.047*	0.031*	0.020*	0.005*	0.009*	0.024*
Upper middle	0.03	0.049*	0.014*	0.032*	0.008*	0.052*	0.000*
Lower middle	0.05	0.067*	0.005*	0.064*	0.009*	0.075*	0.007*
Low	0.04	0.044*	0.019*	0.028*	0.006*	0.024*	0.037*
**Life evaluation**							
All countries	0.24	0.119*	0.024*	0.154*	0.006*	0.117*	0.019*
High income	0.16	0.131*	0.029*	0.105*	0.007*	0.057*	0.011*
Upper middle	0.11	0.099*	0.008*	0.107*	0.005*	0.145*	0.001
Lower middle	0.09	0.064*	0.012*	0.076*	0.005*	0.097*	0.028*
Low	0.1	0.042*	0.009*	0.046*	0.007*	0.050*	0.023*

* p value<5 percent. “Intercept only” model is a multilevel model with no covariates other than the constant. “ICC” stands for “intraclass correlation coefficient”, which is calculated as the ratio of country-level variance versus total variance in the intercept-only model, and can be interpreted as the proportion of total variance attributed to the country level. The key independent variable in model 1 is “social support”; in model 2 the key independent variable is “volunteering”; in model 3 the key independent variable is “social trust”. All models control for age, gender, education, household income, marital status, religiosity, and year dummy variables.

### OLS with country-fixed effects

Table 3 shows standardized coefficients for the PFS from OLS with country-fixed effects. Associations between social support, volunteering, social trust and the PFS are positive and statistically significant across the board. Among the three social capital measures, the link between social support and PFS is the strongest, with a standardized coefficient of 0.267 in the global sample, meaning that having social support increases PFS by about 0.27 standard deviations, holding other covariates in the model constant. Furthermore, there is an increasing return of social support on PFS as national income gets higher: the coefficient is 0.388 (95% CI: 0.315, 0.460) in high-income countries, and 0.223 (0.178, 0.267) in low-income countries. For volunteering (γ_1_ = 0.131) and social trust (γ_1_ = 0.159), the coefficients are similar across income groups. In country-specific analyses, associations of social support on PFS are positive in 95% of the 142 countries, and statistically significant in 75% of those countries. For volunteering the percentages are 86% (positive) and 37% (positive and statistically significant), respectively. For social trust they are 85% (positive) and 35% (positive and statistically significant).

**Table 3:** Standardized coefficients of social capital on positive feelings score (PFS).

	Social support				Volunteering				Social trust			
	*γ_1_*	95% C.I.	R-squared	N	*γ_1_*	95% C.I.	R-squared	N	*γ_1_*	95% C.I.	R-squared	N
All countries	0.267*	(0.241, 0.293)	0.102	214966	0.131*	(0.111, 0.151)	0.094	214966	0.159*	(0.121, 0.197)	0.114	56561
**Income categories**												
High income	0.388*	(0.315, 0.460)	0.077	44039	0.131*	(0.091, 0.170)	0.065	44039	0.143*	(0.059, 0.227)	0.085	10597
Upper middle	0.329*	(0.278, 0.380)	0.125	44796	0.131*	(0.090, 0.172)	0.113	44796	0.174*	(0.072, 0.277)	0.138	9847
Lower middle	0.234*	(0.191, 0.276)	0.125	63854	0.145*	(0.104, 0.187)	0.119	63854	0.168*	(0.089, 0.247)	0.132	20983
Low	0.223*	(0.178, 0.267)	0.073	62277	0.115*	(0.077, 0.154)	0.066	62277	0.154*	(0.090, 0.218)	0.083	15134

* p value<5 percent.

Data source: World Gallup Poll, 2005–2009.

“*γ_1_*” columns indicate standardized coefficients of a social capital measure (social support, volunteering, or trust) on positive feelings score, estimated using OLS with country fixed effects, also controlling for age, gender, education, household income, marital status, religiosity, and year dummy variables.

Data is weighted by sampling weights; robust standard errors clustered at country level are estimated.

Table 4 presents the results for the NFS. In the pooled sample, social support is associated with reduced number of NFS, with standardized coefficient of −0.251 (−0.278, −0.223) in the pooled sample. This means that having social support is associated with a 0.25 standard deviation reduction in the number of negative feelings, holding other covariates constant. Social trust is also negatively associated with NFS in the pooled sample: the coefficient is −0.108 (−0.144, −0.072). Contrary to our hypothesis, volunteering is positively associated with the NFS, though the association is weak: 0.033 (0.012, 0.053) in the pooled sample, non-significant in high income and middle income countries and is 0.042 (0.001, 0.083) in low income countries. The negative association with social support holds across income groups. For social trust, the association is negative across income groups although the coefficient is insignificant in low income countries. In country-specific analysis, associations of social support on NFS are negative in 94% of the 142 countries, and statistically significant in 75% of those countries. For volunteering the percentages are only 39% (negative) and 3.5% (negative and statistically significant), respectively. For social trust they are 79% (negative) and 27% (negative and statistically significant).

**Table 4:** Standardized coefficients of social capital on negative feelings score (NFS).

	Social support				Volunteering				Social trust			
	*γ_1_*	95% C.I.	R-squared	N	*γ_1_*	95% C.I.	R-squared	N	*γ_1_*	95% C.I.	R-squared	N
All countries	−0.251*	(−0.278, −0.223)	0.069	214966	0.033*	(0.012, 0.053)	0.059	214966	−0.108*	(−0.144, −0.072)	0.077	56561
**Income categories**												
High income	−0.360*	(−0.481, −0.238)	0.059	44039	0.017	(−0.025, 0.058)	0.046	44039	−0.194*	(−0.289, −0.100)	0.043	10597
Upper middle	−0.334*	(−0.382, −0.285)	0.076	44796	0.047	(−0.002, 0.096)	0.062	44796	−0.132*	(−0.192, −0.071)	0.087	9847
Lower middle	−0.222*	(−0.251, −0.193)	0.076	63854	0.024	(−0.019, 0.068)	0.068	63854	−0.062*	(−0.107, −0.016)	0.085	20983
Low	−0.195*	(−0.240, −0.150)	0.062	62277	0.042*	(0.001, 0.083)	0.054	62277	−0.085	(−0.172, 0.001)	0.051	15134

Notes:

* p value<5 percent.

Data source: World Gallup Poll, 2005–2009.

“*γ_1_*” columns indicate standardized coefficients of a social capital measure (social support, volunteering, or trust) on negative feelings score, estimated using OLS with country fixed effects, also controlling for age, gender, education, household income, marital status, religiosity, and year dummy variables.

Data is weighted by sampling weights; robust standard errors clustered at country level are estimated.

Table 5 shows the results for the GLE. In the pooled sample, the standardized coefficient of social support is positive and significant: 0.291 (0.265, 0.316), meaning that having social support is associated with 0.29 standard deviation increase in GLE, holding other covariates constant. The coefficients are 0.077 (0.059, 0.094) and 0.118 (0.075, 0.162) for volunteering and social trust respectively. In analyses stratified by country-level income, the positive associations with social support, volunteering or social trust hold across all country-level incomes, except that the coefficient of social trust on GLE is insignificant in low income countries. The associations are largest in high-income countries and smallest in low-income countries. In country-specific analyses, the associations of social support on GLE are positive in 96% of the countries, and statistically significant in 84% of those countries. For volunteering, the percentages are 76% (positive) and 22% (positive and statistically significant) respectively. For social trust they are 71% (positive) and 36% (positive and statistically significant).

**Table 5:** Standardized coefficients of social capital on life evaluation (GLE).

	Social support				Volunteering				Social trust			
	*γ_1_*	95% C.I.	R-squared	N	*γ_1_*	95% C.I.	R-squared	N	*γ_1_*	95% C.I.	R-squared	N
All countries	0.291*	(0.265, 0.316)	0.258	214966	0.077*	(0.059, 0.094)	0.246	214966	0.118*	(0.075, 0.162)	0.232	56561
**Income categories**												
High income	0.402*	(0.337, 0.468)	0.220	44039	0.105*	(0.073, 0.136)	0.206	44039	0.165*	(0.069, 0.260)	0.116	10597
Upper middle	0.390*	(0.341, 0.440)	0.178	44796	0.072*	(0.032, 0.112)	0.160	44796	0.126*	(0.060, 0.191)	0.166	9847
Lower middle	0.285*	(0.242, 0.327)	0.137	63854	0.069*	(0.029, 0.110)	0.123	63854	0.142*	(0.063, 0.220)	0.166	20983
Low	0.207*	(0.174, 0.240)	0.141	62277	0.064*	(0.036, 0.093)	0.129	62277	0.058	(−0.042, 0.158)	0.150	15134

Notes:

* p value<5 percent.

Data source: World Gallup Poll, 2005–2009.

“*γ_1_*” columns indicate standardized coefficients of a social capital measure (social support, volunteering, or trust) on life evaluation, estimated using OLS with country fixed effects, also controlling for age, gender, education, household income, marital status, religiosity, and year dummies.

Data is weighted by sampling weights; robust standard errors clustered at country level are estimated.

## Discussion

Our study explores the associations between subjective well-being and social capital in 142 countries spanning low, middle and high income countries. In the pooled analysis, we find evidence of significant associations between measures of social support, social capital and better subjective well-being, after adjusting for age, gender, religiosity, marital status, education, household income, and year of interview. Individuals with social support, who participate in volunteering activities, and with high levels of interpersonal trust are more likely to have higher life evaluations and higher positive feeling scores, compared to peers with less civic involvement, no social support and lower social trust.

As predicted, the association between social support and life evaluation is strongest in high-income countries and weakest in low-income countries. We find similar patterns for the association between life evaluation, volunteering and social trust. PFS and NFS follow a similar pattern with stronger associations in high-income countries and lower associations in low-income countries.

Country-level analyses for social support and trust portray a similar association although not for every country. However, associations between volunteering and SWB are not consistent. Actually, most associations between volunteering and negative feelings are not significant across countries. In addition, the association between volunteering and the negative feeling score is positive in low-income countries. An explanation may be that while volunteering may have a high social value in Western countries, it may have a different and smaller social value in other countries [60]. The motives for volunteering may differ as well among countries. For instance, Ziemek found that a main motivation for volunteers in countries with high public spending was the investment in their own human capital instead of for altruistic reasons [61]. In South Africa, within a context of extreme poverty, family members of patients with HIV/AIDS experienced negative feelings (sadness, anger, frustration) as they cared for their loved ones [62].

Our paper also suggests that the affective component of subjective well-being is comprised of two distinctive constructs rather than of opposite sides of the same continuum. For instance, in low-income countries the association between social support and positive feelings is stronger than the association between social support and negative feelings. Concerning volunteering, there is a consistent association with positive feeling around the world whereas the association with negative feelings most often not significant. Social trust is more strongly associated with positive feelings than with negative feelings, particularly in middle and low-income countries.

A major strength of this study is the use of a harmonized cross-national survey in which all regions of the world are represented. A limitation rests on the cross sectional nature of the study prohibiting us from understanding the causal or temporal direction of associations. Although we have adjusted for observable potential confounders in our models, other unobservable confounders may remain. Reverse causation is another concern. It is possible that people who are more satisfied with their lives are volunteering more or have more people to count on in case of need. There is evidence on the bidirectional association between subjective well-being and a variety of positive outcomes, including social support [63]. Finally, our measure of life satisfaction may measure with error the actual cognitive component of SWB, by asking respondents to evaluate their *present*, rather than their *whole*, life. This is a potentially serious confound, as temporal specificity of this item may affect response patterns. Before 2009 the Gallup World Poll also asked respondents to evaluate their life five years ago, and to guess where they would stand in the future, using the same ladder scale from 0 to 10. We constructed a measure using the average of answers to the three questions and it was highly correlated with the GLE on current life (correlation coefficient is 0.88). Regressions using this measure generate very similar results as the regressions using GLE on current life. Since the question on evaluating life five years ago was not asked in most countries in 2009, we employ the measure of GLE on current life. Furthermore, we have controlled for religiosity and marital status in the analyses. This is a conservative strategy since religious activities may be a substantial source of social capital. Analysis with and without religiosity are not very different suggesting that religiosity does not influence the link between social capital and SWB.

Our study has limited capacity to assess the mechanisms through which social capital might influence subjective well-being. It is challenging to pinpoint the pathways in a cross-national study because of diverse country specific characteristics. Differences in culture might influence the relationship between social capital and SWB. It is also plausible that there may be systematic differences in the meaning attributed to the response items in different languages or there may be reporting biases that are culturally-based. Pathways linking social capital to subjective well being might include behavioral pathways related to increased social interactions, collective actions that might increase a range of public goods and services that would increase subjective well being and cognitive processes that lead directly to well being. We will need to leave the analysis of such pathways to other investigators with other studies.

The importance of contextual variables cannot be underestimated. Social and economic contexts may shape patterns of social capital however; the identification of such contextual conditions is beyond the scope of this paper. The main objective of this analysis was to extend the association between subjective well-being and social support, volunteering and social trust in low and middle-income countries and examine whether the affective component of subjective well-being behaves similarly in relation to proxy measures of social capital world wide.

We conclude that having somebody to count on in case of need and high levels of social trust are associated better life evaluations and more positive feelings and an absence of negative feelings in most countries on the world. The importance of social capital has also been echoed in a related paper by Kumar et. al. [64]. Using the Gallup data, Kumar and colleagues showed that social support positively affected self-reported health in most of the countries across the world. Their results indicate that the strong association between social capital and health is not restricted to high-income countries but extends across many geographical regions regardless of their national-income level. Associations, however, are stronger for high and middle-income countries. Volunteering is also associated with better life evaluations and a higher frequency of pleasant emotions. There is not an association, however, between volunteering and experiencing negative feelings, except in low-income countries. Finally, we present evidence that the two affective components of subjective well-being behave differently in relation to different proxies of social capital and social support across countries.

Further research must identify the reasons for the differing associations so that we can understand whether differing associations are the result of cultural differences in the meaning and interpretation of the questions or reflect more genuine differences. As we move closer to considering ways to improve subjective well being, it will be important to understand the causal ties between social capital and subjective well-being as well as the options available to increase social capital. Policy interventions often rely on modifying the mediating conditions in such causal pathways Denier and Chan [2] provide an excellent review of the literature that establishes casual mechanism between subjective well-being and objective measures of health. In this study, we have provided a number of additional examples that link health with subjective well-being.
